# The effect of psychological flexibility, self-compassion, and mindfulness on mental health among university students

**DOI:** 10.3389/fpsyg.2026.1757032

**Published:** 2026-03-17

**Authors:** Yunus Sahinler, Mustafa Deniz Dindar, Mustafa Can Koc

**Affiliations:** 1 Faculty of Sports Sciences, Istanbul Gelişim University, Istanbul, Türkiye; 2 Directorate of Sports Sciences Application and Research Center, Istanbul Gelisim University, Istanbul, Türkiye; 3 Faculty of Sports Sciences, Canakkale Onsekiz Mart University, Canakkale, Türkiye

**Keywords:** mental health, mindfulness, psychological flexibility, self-compassion, university students

## Abstract

**Background:**

Although studies have been conducted on psychological flexibility, self-compassion and mindfulness, the effects of these factors on the mental health of university students have not yet been sufficiently researched.

**Objective:**

This study seeks to investigate the effects of psychological flexibility, self-compassion, and mindfulness on mental health among university students in Türkiye.

**Methods:**

The data were analyzed using SPSS 26.0, AMOS, and the Hayes PROCESS macro. Descriptive statistics and Pearson correlations were first calculated. The hypothesized relationships were tested using Hayes PROCESS Model 4 multiple mediation analysis with 5,000 bootstrap samples to estimate direct and indirect effects.

**Results:**

The results revealed a high and positive correlation between psychological flexibility (pf) and mindfulness (m) (ρ = 0.712, *p* < 0.01). Pf was also shown to have strong and significant correlations with self-compassion (s) (ρ =0.762, p < 0.01) and mental health (mh) (ρ = 0.668, *p* < 0.01). Multiple regression analysis revealed a significant direct relationship between mental health and psychological flexibility (*b* = 0.1769, se = 0.0536, *p* < 0.001).

**Conclusions:**

The study demonstrate that psychological flexibility has a significant and complex impact on college students' mental health. People are better at handling stress, more self-compassionate, and more attentive, as seen by the strong and favorable correlations between psychological flexibility, mindfulness, self-compassion, and mental health.

## 1 Introduction

A university education is a crucial time for individuals to build their identities, independence, and coping mechanisms in addition to their academic abilities. Students experience a variety of stressors during this process, including pressure to perform well academically, issues in social interactions, financial hardships, future anxiety, and being apart from family (Bayram and Bilgel, 2008). Although these elements may have a detrimental impact on a person's mental health, psychology, cognitive awareness, and internal resources can help shield a person from these challenges. Research has started to focus more on the impact of personal traits including psychological flexibility, self-compassion, and mindfulness on college students' mental health in recent years (Goodman et al., 2018; Keng et al., 2011). According to Kashdan and Rottenberg (2010), psychological flexibility is the ability of a person to adjust and preserve emotional equilibrium in the face of trauma, stress, failure, or other unfavorable life events. According to Rostampour Brenjestanaki et al. (2020), psychological flexibility is the ability to accept our ideas and feelings and act on long-term ideals rather than short-term impulses, thoughts, and feelings that are frequently connected to experiencing avoidance and a strategy of controlling undesirable internal events. This idea speaks to a person's capacity to not only overcome obstacles but also develop by taking lessons from them (Bonanno, 2004). Pupils with high psychological flexibility can draw a more psychologically resilient profile because they can view stressful experiences as learning opportunities rather than as threats (Tugade and Fredrickson, 2004). Psychological flexibility is a valuable tool for students to preserve their emotional balance and academic success when there are several stressors, like the university setting (Pidgeon et al., 2014). Self-compassion involves being kind and understanding toward oneself in instances of pain or failure, perceiving one's experiences as part of the larger human experience, and evaluating negative feelings with a balanced awareness without exaggerating them (Neff, 2003a). According to (Neff 2003a) model, self-compassion consists of three components: self-kindness, common humanity, and mindfulness. A high degree of self-compassion helps individuals cope with negative feelings and reduces depression, anxiety, and stress (MacBeth and Gumley, 2012). Particularly in case of academic failures or social rejection, student's self-critical judgments can be replaced by a constructive internal dialogue through the self-compassion mechanism. This is a crucial factor by which psychological wellbeing is promoted (Zessin et al., 2015). Mindfulness is defined as one's conscious and deliberate attention to the present moment and its acceptance without judgment (Kabat-Zinn, 2003). Practicing mindfulness, individuals may notice their mental processes more clearly, distance their thoughts, and regulate their emotional reactions in a healthier way (Brown and Ryan, 2003). In this respect, mindfulness is a powerful tool for reducing the effects of stress, providing mental clarity, and increasing psychological resilience. Studies conducted on university students have shown that mindfulness practices significantly lower levels of anxiety and depression along with a favorable impact on academic performance (Shapiro et al., 2008; de Vibe et al., 2018). Taken together, psychological flexibility, self-compassion, and mindfulness are shown to be innate abilities that contribute to mental health and support each other. Mindfulness helps people notice their internal experiences and respond rather than react, self-compassion allows people to judge themselves with such mindfulness, and psychological flexibility allows people to adapt to challenges with all these internal resources (Hayes et al., 2006; Neff and Dahm, 2015). In this regard, assessing these three psychological resources collectively offers a more comprehensive and illuminating approach to university students' mental health. Recent models in contextual behavioral science conceptualize psychological flexibility as a meta-process that enables individuals to regulate internal experiences through acceptance and value-based action (Hayes et al., 2006). Within this framework, mindfulness and self-compassion are considered complementary mechanisms that operationalize psychological flexibility in daily life. Mindfulness facilitates non-judgmental awareness of thoughts and emotions, whereas self-compassion reduces self-criticism and promotes adaptive emotion regulation. Empirical studies indicate that these processes jointly contribute to lower psychological distress and higher wellbeing among university students (Keng et al., 2011; MacBeth and Gumley, 2012). Therefore, examining these variables within an integrated model provides a more comprehensive understanding of student mental health. Focusing on the investigation of the impacts of degrees of psychological flexibility, self-compassion, and mindfulness on university students' mental health, this study seeks to better understand the protective psychological elements that are likely to improve their mental wellbeing. Although previous studies have examined psychological flexibility, self-compassion, and mindfulness as independent predictors of mental health, limited research has addressed these constructs within a single integrative mediation model among Turkish university students. Most existing studies focus either on mindfulness-based interventions or on self-compassion as isolated protective factors, leaving the interactive and hierarchical relationships between these variables insufficiently explored. The present study contributes to the literature by simultaneously testing mindfulness and self-compassion as parallel mediators in the relationship between psychological flexibility and mental health using Hayes' PROCESS Model 4. This approach enables a more nuanced understanding of whether psychological flexibility influences mental health directly or primarily through compassion- and awareness-based mechanisms within a non-clinical university population in Türkiye.

## 2 Materials and methods

### 2.1 Research questions and hypotheses

**Research Questions**

This study sought to answer the following research questions:

1. Is psychological flexibility significantly associated with the mental health of university students?
2. Do mindfulness and self-compassion mediate the relationship between psychological flexibility and mental health?
3. What is the relative contribution of mindfulness and self-compassion in explaining mental health outcomes?

**Hypotheses**

Based on the theoretical framework and previous empirical findings, the following hypotheses were formulated:

**H1:** Psychological flexibility will have a significant direct positive effect on mental health.

**H2:** Psychological flexibility will have a significant positive effect on mindfulness.

**H3:** Psychological flexibility will have a significant positive effect on self-compassion.

**H4:** Mindfulness will have a significant positive effect on mental health.

**H5:** Self-compassion will have a significant positive effect on mental health.

**H6:** Mindfulness and self-compassion will mediate the relationship between psychological flexibility and mental health.

### 2.2 Objectives and significance of the research

This study's primary goal is to investigate how university students' mental health is impacted by psychological flexibility, self-compassion, and mindfulness levels. The study examined if psychological flexibility has a direct or indirect impact on mental health as well as whether internal resources like self-compassion and mindfulness mediate this link. In this case, the associations between variables were thoroughly investigated using multiple mediation analysis using (Hayes 2022) PROCESS Model 4. The selection of mindfulness and self-compassion as mediators was grounded in Acceptance and Commitment Therapy (ACT) and self-regulation theories. Psychological flexibility is conceptualized as an overarching meta-skill that facilitates adaptive emotion regulation and value-based behavior. However, empirical models suggest that this effect on mental health is largely transmitted through two mechanisms: (a) mindful awareness of internal experiences, which reduces experiential avoidance, and (b) a compassionate stance toward the self, which mitigates self-criticism and rumination. Therefore, mindfulness and self-compassion were theoretically positioned as parallel mediators linking psychological flexibility to mental health outcomes.

### 2.3 Population and sample

The population of the study consisted of university students enrolled in higher education institutions in Istanbul, Türkiye. The sample comprised 526 students from Istanbul Gelişim University and Istanbul University. A convenience sampling method was employed. Of the participants, 378 were female (71.9%) and 148 were male (28.1%). Regarding age distribution, 36.8% (*n* = 194) were between 18-24 years, 39.4% (*n* = 207) were between 25-29 years, and 23.8% (*n* = 125) were 30 years or older. In terms of educational status, 48.8% (*n* = 257) were undergraduate students, 27.4% (*n* = 144) were postgraduate students, and 23.8% (n = 125) were associate degree students. Participants were reached through institutional e-mail announcements and student social media groups. Participation was voluntary and anonymous, and no incentives were provided.

### 2.4 Data collection tools

Data was collected via an online survey using Google Forms. Prior to participation, an informed consent form was provided outlining the distribution, distribution method, confidentiality of responses, and the basis for volunteer participation. Only those who consented were allowed to proceed with the survey. Completing the survey took approximately 10-12 min. No personally identifiable information was collected, and all responses were stored anonymously. The study protocol was reviewed and approved by the Siirt University Ethics Committee (Decision No: 8696, dated 10.03.2025). The research was conducted in accordance with the Helsinki Declaration and relevant national regulations.

### 2.5 Personal information form

It consists of 4 questions in total, including gender, age, educational status, and a history of receiving support for mental health.

#### 2.5.1 Acceptance and Action Form-II (AAP-II)

The Acceptance and Action Form-II (AAP-II) is aimed at measuring one's psychological flexibility degree. Psychological flexibility is one's ability to continue to focus on important goals in life together with acceptance of negative feelings and thoughts rather than suppressing them. This scale assesses how individuals cope with challenging feelings and thoughts and how they impact daily lives. KEF-II was created by Bond et al. (2011), and Yıldırım et al. (2017) translated it into Turkish. It is one-dimensional and assesses psychological flexibility directly. The seven items on the scale are rated on a 5-point Likert scale: (1): Strongly disagree, (2): disagree, (3): Undecided, (4): agree, and (5): strongly agree. The scale's items 1, 2, 4, and 5 have reverse codes. Reverse-coded items are fixed and added up to determine the final score. Higher scores are indicative of greater psychological flexibility. KEF-II's reliability assessments demonstrate its great internal consistency: Cronbach's Alpha (internal consistency coefficient) is 0.83, and test-retest reliability is 0.79. The KEF-II is regarded as one of the most accurate measures of psychological flexibility.

#### 2.5.2 Short Form of Self-Compassion Scale (SCS-SF) Scale

Designed by (Neff 2003b) and translated into Turkish by Yildirim et al., it assesses people's levels of self-compassion and has a one-dimensional design. Twelve items make up the scale, and a 5-point Likert-type rating system is employed: (1): Never, (2): Seldom, (3): Occasionally, (4): Frequently, and (5): Always. Reverse-coded items include 1, 4, 8, 9, 10, and 11. The reverse-coded items are corrected and added up to determine the final score. High scores show that the person has a strong degree of self-compassion. The scale's psychometric qualities are strong, according to validity and reliability analyses. The internal consistency coefficient (Cronbach's Alpha) is 0.85 and test-retest reliability is 0.81.

#### 2.5.3 Cognitive and affective mindfulness scale

The Cognitive and Affective Mindfulness Scale is aimed at measuring one's mindfulness degrees. Developed by Feldman et al. (2007) and adapted into Turkish by Çatak (2012), CAMS-R consists of 10 items and has a one-dimensional structure. It is rated using a 4-point Likert-type rating (1 = Never, 2 = Rarely, 3 = Sometimes, 4 = Always). The total score of the scale is calculated by summing all items, and higher scores indicate a higher degree of mindfulness. The internal consistency coefficient of the scale (Cronbach's Alpha) was found to be 0.77.

#### 2.5.4 General Health Questionnaire-12 (GHQ-12) Scale

General Health Questionnaire-12 (GHQ-12) is a scale developed to assess the general mental health status of individuals as well as to identify degrees of psychological distress. GHQ-12 assesses individuals' recent feelings and thoughts regarding stress, anxiety, depression, loss of self-confidence, and daily functioning. The scale was developed by Williams and Goldberg (1988) and adapted into Turkish by Kiliç et al. (1997). The scale consists of 12 items and a 4-point Likert-type rating system is used: (1): Not at all, (2): No more than usual, (3): More than usual, (4): Much more than usual. Items consist of positively and negatively worded questions while some items are reverse coded. The total score is calculated once reverse-coded items are corrected and summed, and higher scores indicate lower degrees of mental health. Validity and reliability analyses of the GHQ-12 reveal that the scale has strong psychometric properties. The internal consistency coefficient (Cronbach's Alpha) is 0.82 and test-retest reliability is 0.79. The GHQ-12 is widely used in mental health research to measure stress and anxiety and to assess psychological wellbeing in non-clinical populations. In addition to measuring mental health, the scale is also used to assess the need to apply mental health services.

### 2.6 Analysis of data

The data of the study were analyzed using SPSS 26.00, HAYES Process, and AMOS statistical package programs. While testing the research data, a significance level of 0.05 was taken into consideration. Frequency (f), percentage (%), and weighted average (x) values were used when analyzing the descriptive data. Once the frequencies were taken following the data analysis, a normality test and reliability analysis were performed for the reliability of the data. The normality analysis revealed that the data showed a normal distribution (±,980). Cronbach alpha and confirmatory factor analysis was performed for the reliability and validity of the scales. Correlations between variables were analyzed using Pearson correlation analysis with the effects between variables tested using Hayes' PROCESS macro (Model 4). Regression tests revealed that demographic characteristics (gender, age, educational attainment, and mental health support) had little to no significant impact on the outcome variables, hence the study did not account for these when evaluating the model. The mediating roles of mindfulness (BM) and self-compassion (SCS) in the relationship between psychological flexibility (PF) and university students' mental health (MS) were examined in the data analysis.

## 3 Results

There are a total of 526 participants. In terms of gender distribution, 71.9% are female participants (*n* = 378), and 28.1% are male participants (*n* = 148). The largest age group, with 207 individuals (39.4%), is the 25-29 age bracket, according to the age distribution. One hundred and ninety four participants (36.8%) in the 18–24 age group and 125 individuals (23.8%) in the 30+ age group come next. Regarding educational status, nearly half of the participants hold a bachelor's degree (*n* = 257, 48.8%), 27.4% have a postgraduate degree (*n* = 144), and 23.8% have an associate degree (n = 125).

When asked “Have you received mental health support?” 31.4% (*n* = 165) of the participants responded as “yes” and 68.6% (n = 361) responded as “no”.

Table 2 shows that the distributions of all variables included in the study are within acceptable ranges. The skewness values for the variables Psychological Resilience (PF), Mindfulness (M), Self-Compassion (SC), and Mental Health (MH) range from 0.258 to 0.435, and the basis values range from 0.371 to 0.854. These values, ranging from −1 to +1, indicate that the data conform to a normal distribution. The Cronbach's α values, adjusted for the internal coefficients of the scales, were determined to range between 0.86 and 0.90. Accordingly, α = 0.89 for Psychological Resilience performance, α = 0.88 for Mindfulness performance, α = 0.90 for Self-Compassion performance, and α = 0.86 for Mental Health performance (Table 1).

**Table 1:** Distribution characteristics of the variables as part of research.

**Variable**	**Min**	**Max**	**Skewness**	**Kurtosis**	**Cronbach's α**
Psychological Flexibility (PF)	1.00	4.00	0.258	0.645	0.89
Mindfulness (M)	1.00	5.00	0.365	0.371	0.88
Self-Compassion (SC)	1.20	5.00	0.412	0.402	0.90
Mental Health (MH)	1.00	5.00	0.435	0.854	0.86

**Table 2:** Means, standard deviations, and correlations of variables (n = 526).

**Variable**	**M**	**SD**	**1**	**2**	**3**	**4**
1. Psychological Flexibility (PF)	2.097	0.517	-	0.712^**^	0.762^**^	0.668^**^
2. Mindfulness (M)	2.985	0.687		-	0.730^**^	0.669^**^
3. Self-Compassion (SC)	3.012	0.701			-	0.751^**^
4. Mental Health (MH)	2.547	0.368				-

Table 2 presents the mean, standard deviation, and Pearson correlation coefficients for the research variables. The findings show positive and significant relationships among all variables. A high level of correlation was found between psychological resilience and mindfulness (*r* = 0.712, *p* < 0.01). Similarly, psychological resilience was strongly correlated with self-compassion (r = 0.762, p < 0.01) and mental health (*r* = 0.668, *p* < 0.01). The mindfulness variable showed significant and high-level correlations with self-compassion (*r* = 0.730, *p* < 0.01) and mental health (*r* = 0.669, *p* < 0.01). The strongest correlation among the variables was observed between self-compassion and mental health (*r* = 0.751, *p* < 0.01).

The PROCESS macro Model 4 proposed by (Hayes 2022) was used to evaluate the mediating role of self-compassion (SC) and mindfulness (M) under the effect of psychological flexibility (PF) on the mental health (MH) of university students in Table 3. The analyses were conducted on 526 participants, and the bootstrap sample number was determined as 5,000. The effect of psychological flexibility on mindfulness was found to be quite strong (*B* = 0.7267, *SE* = 0.0316, *t* = 23.02, *p* < 0.001). The reliability of the relevant relationship was high with the confidence interval [0.6647, 0.7888]. The effect of psychological flexibility on self-compassion was also highly significant (*B* = 1.0774, *SE* = 0.0379, *t* = 28.46, *p* < 0.001). The confidence interval was [1.0030, 1.1517], indicating that the effect size was quite strong and significant. The direct effect of psychological flexibility on mental health was also found to be statistically significant (*B* = 0.1769, *SE* = 0.0536, *t* = 3.30, *p* < 0.001). The confidence interval was [0.0716, 0.2822], indicating that this relationship was not random but pointed to a significant model explanation. In addition, mindfulness also stood out as a significant predictor of mental health (*B* = 0.1801, *SE* = 0.0482, *t* = 3.74, *p* < 0.001). The confidence interval [0.0854, 0.2747] supported the statistical reliability of this relationship. The effect of self-compassion on mental health was highly significant (*B* = 0.4978, *SE* = 0.0402, *t* = 12.39, *p* < 0.001). The confidence interval [0.4189, 0.5767] strengthened the statistical significance of this effect.

**Table 3:** Regression results showing the relationship between psychological flexibility, mindfulness, self-compassion, and mental health.

**Variable**	***B***	***SE***	***t***	***p***	**95% GA**
M ~ PF	0.7267	0.0316	23.02	< 0.001	[0.6647, 0.7888]
SC ~ PF	1.0774	0.0379	28.46	< 0.001	[1.0030, 1.1517]
MH~ PF	0.1769	0.0536	3.30	< 0.001	[0.0716, 0.2822]
MH ~ M	0.1801	0.0482	3.74	< 0.001	[0.0854, 0.2747]
MH ~ SC	0.4978	0.0402	12.39	< 0.001	[0.4189, 0.5767]

As shown in Table 4, the direct effect of psychological flexibility on mental health was significant (*B* = 0.1769, *SE* = 0.0536, *p* = 0.001). Indirect effects were realized through self-compassion and mindfulness. Confidence intervals of all bootstraps showed that indirect effects were statistically significant. These results indicate that the effect of psychological flexibility on mental health also occurs indirectly through self-compassion and mindfulness.

**Table 4:** Direct and indirect effects on the relationships between psychological flexibility, mindfulness, self-compassion, and mental health.

**Indirect**	**Effect**	**BootSE**	**95% GA**
**Total**	0.6672	0.0762	[0.5205, 0.8169]
**PF** ** → M** ** → MH**	0.1309	0.0498	[0.0401, 0.2365]
**PF** ** → SC** ** → MH**	0.5363	0.0642	[0.4106, 0.6631]

**Figure 1:**
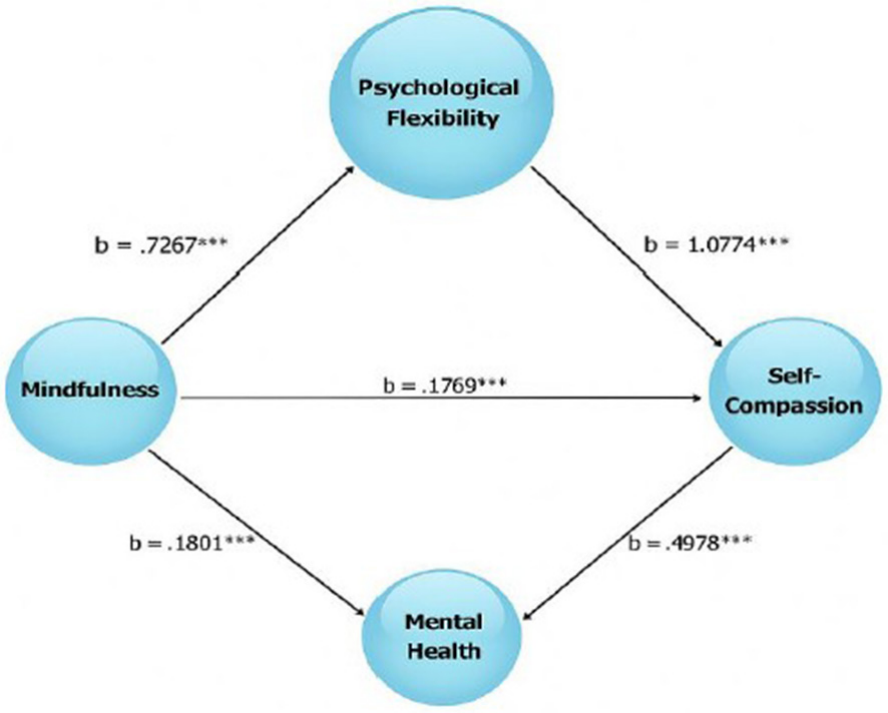
shows the structure of the mediation model based on Model 4 used in the study. ***** is used for p < 0.001; ** is used for *p* < 0.01. All paths are significant and the model explanatory power is high. The asterisk symbol, used to indicate relationship level, is generally used in bulleted lists to highlight important criteria, defining elements, or key points that need to be emphasized (such as commitment, trust, communication, shared interests, etc.).

The mediator variables M (Mindfulness) and SC (Self-Compassion) were included in the model shown in Figure 1. All paths in the model were found to be statistically significant. Psychological flexibility had a highly significant and positive effect on mindfulness (PF → M (b = 0.7267). The effect of psychological flexibility on self-compassion was found to be quite strong (PF → SC (b = 1.077). Mindfulness was found to be a significant predictor of mental health (SC → MH (b = 0.4978).

The serial mediation analysis demonstrated that Psychological Flexibility (PF) significantly predicted Mindfulness (M) (a1 = 0.7267, p < 0.001), and Mindfulness, in turn, significantly predicted Self-Compassion (SC) (a2 = 1.0774, p < 0.001). Both Mindfulness and Self-Compassion were significant positive predictors of Mental Health (MH) (b1 = 0.1801, *p* < 0.001; b2 = 0.4978, *p* < 0.001). Importantly, the direct effect of Psychological Flexibility on Mental Health remained statistically significant (c′ = 0.1769, *p* < 0.01), indicating partial mediation. These findings support a sequential mediation model in which Psychological Flexibility enhances Mental Health. Specifically, higher Psychological Flexibility is associated with greater Mindfulness, which fosters increased Self-Compassion, ultimately contributing to better Mental Health. Thus, the relationship between Psychological Flexibility and Mental Health operates both directly and indirectly through a chain of interrelated psychological processes involving Mindfulness and Self-Compassion.

**Figure 2:**
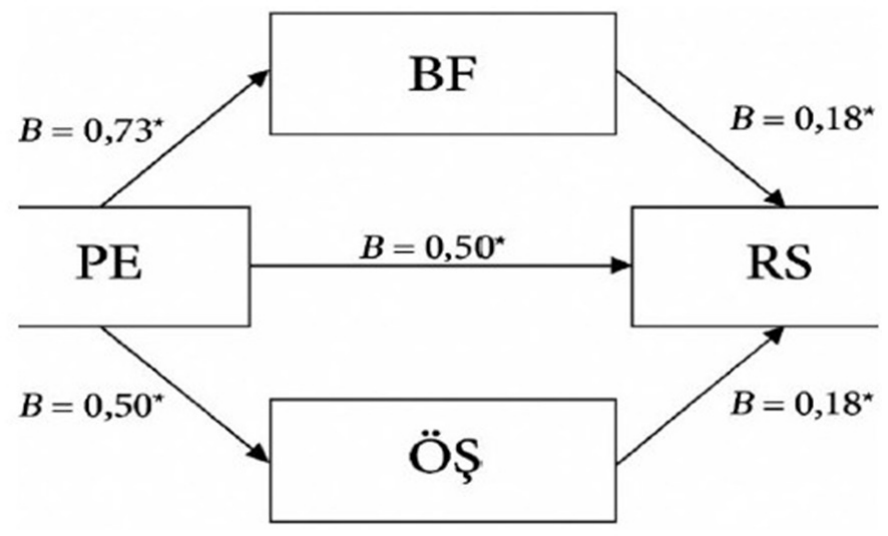
Includes a diagram model showing the impact of psychological flexibility on the mental health of university students through self-compassion and mindfulness. The asterisk symbol, used to indicate relationship level, is generally used in bulleted lists to highlight important criteria, defining elements, or key points that need to be emphasized (such as commitment, trust, communication, shared interests, etc.).

The parallel mediation analysis based on PROCESS Model 4 revealed that PF had a significant direct effect on MH (B = 0.50, *p* < 0.001). In addition, PF significantly predicted both mediators, M (B = 0.73, *p* < 0.001) and SC (*B* = 0.50, *p* < 0.001). Both M and SC were significant positive predictors of MH (*B* = 0.18, *p* < 0.01 for each path) (Figure 2). These findings indicate that PF influences MH not only directly but also indirectly through M and SC. The persistence of a significant direct path alongside significant indirect paths suggests a pattern of partial mediation. Overall, the results support the proposed mediation structure and demonstrate that both mediators play meaningful roles in explaining the relationship between PF and MH.

## 4 Discussion

The purpose of this study was to determine the relationship between university students' psychological flexibility, self-compassion, and mindfulness and their mental health. The results demonstrated that psychological flexibility influences mental health directly as well as indirectly through mindfulness and self-compassion. This result is in line with several studies in the literature and highlights the necessity of comprehensive interventions to enhance university students' psychological resilience (Kashdan and Rottenberg, 2010; Hayes et al., 2006; Pidgeon et al., 2014).

The study discovered a strong correlation between psychological flexibility and mindfulness (B = 0.7267, *p* < 0.001) and self-compassion (B = 1.0774, *p* < 0.001). This finding demonstrates that people can learn to approach themselves in stressful situations with greater understanding, acceptance, and mindfulness (Bonanno, 2004; Troy and Mauss, 2011). According to earlier research, psychological flexibility improves a person's ability to control their emotions and is linked to favorable psychological outcomes (Southwick and Charney, 2012; Chen and Bonanno, 2020).

Self-compassion is one of the study's most compelling mediation findings. A very strong predictor of mental health was found to be self-compassion (*B* = 0.4978, *p* < 0.001). This demonstrates that people can lower their stress and depression levels when they tackle their challenges with empathy rather than condemnation (Neff, 2003a; MacBeth and Gumley, 2012). Furthermore, it has been demonstrated that Mindful Self-Compassion programs created by Neff and Germer (2013) are successful in lowering symptoms like loneliness, social anxiety, and burnout, particularly in college students. Accordingly, self-compassion has a healing effect on interpersonal relationships in addition to raising levels of mindfulness (Tóth-Király et al., 2021).

**Figure 3:**
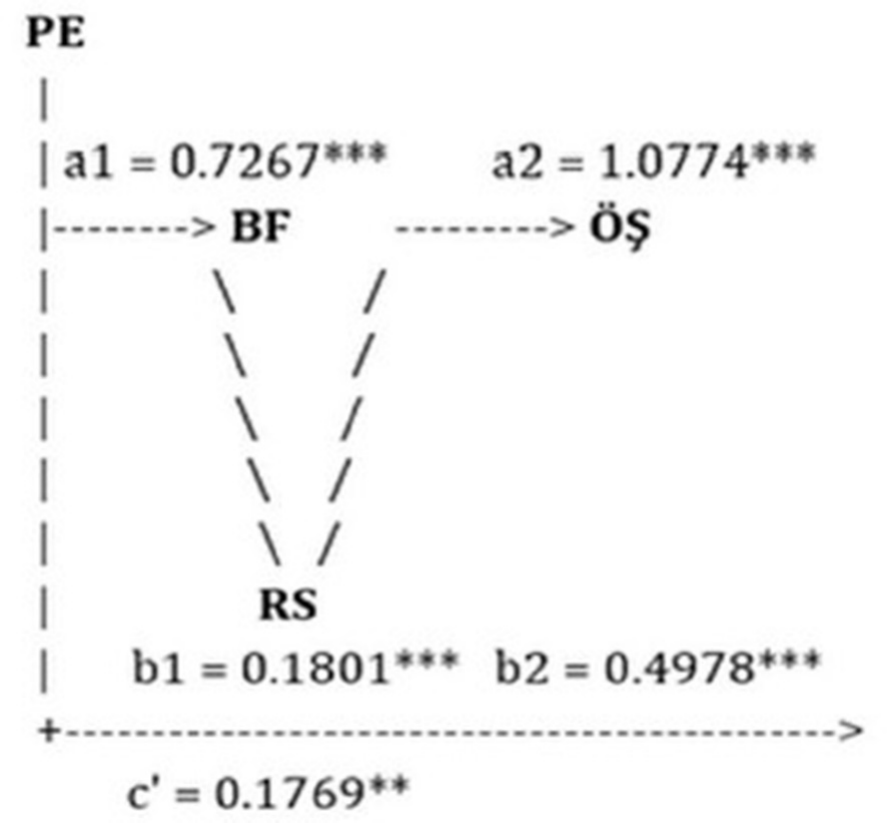
Multiple mediation model showing the relationships between psychological flexibility, mindfulness, self-compassion and mental health. The asterisk symbol, used to indicate relationship level, is generally used in bulleted lists to highlight important criteria, defining elements, or key points that need to be emphasized (such as commitment, trust, communication, shared interests, etc.).

This study also found that mindfulness was a significant predictor of mental health (*B* = 0.1801, *p* < 0.001) (Figure 3). Numerous studies have documented the benefits of mindfulness in lowering psychiatric symptoms by allowing people to approach their present circumstances with an accepting and open mindset (Keng et al., 2011; Brown and Ryan, 2003; Shapiro et al., 2008). Specifically, mindfulness-based therapies were found to dramatically lower levels of stress, anxiety, and depression while also increasing overall life satisfaction in long-term research of university students by de Vibe et al. (2018).

The study's indirect effect analyses (Hayes PROCESS Model 4) reveal that self-compassion (B = 0.5363) and mindfulness (*B* = 0.1309) play a major role in mediating the impact of psychological flexibility on mental health. These results provide credence to the notion that psychological flexibility is intricately linked to emotion regulation and mindfulness abilities, in addition to being a cognitive strategy (Creswell and Lindsay, 2014). In this regard, it is believed that psychoeducation programs that aim to increase psychological resilience should incorporate not just cognitive frameworks but also mindfulness-based and compassion-based techniques.

This study also confirmed the strong correlation between self-compassion and mindfulness, which (Neff 2003a) highlighted (*r* =0.730). This indicates that when openness to one's inner experiences is coupled with both mental and emotional acceptance, the impact on mental health is amplified (Germer and Neff, 2019). Additionally, this is in line with research showing that intervention trials that combine mindfulness and self-compassion training (e.g., MSC, MBCT) are more successful (Kuyken et al., 2016; Kirby J. N. et al., 2017).

In summary, the findings of this study highlight the protective role of psychological flexibility, mindfulness, and self-compassion in university students' mental health. The results suggest that strengthening these internal resources may help students cope more effectively with academic and social stressors. Therefore, university-based preventive programs focusing on mindfulness and compassion-based skills may contribute to improved psychological wellbeing in non-clinical student populations.

## 5 Conclusions

In summary, this study has demonstrated the importance of personal resources including psychological flexibility, self-compassion, and mindfulness in promoting college students' mental health.

It is advised that educational establishments offer structured curricula that enable learners to develop these psychological competencies. Students' psychological wellbeing will rise with interventions that specifically target the development of stress management, emotional resilience, and self-regulation abilities. It is advised that future research use experimental and longitudinal designs to more thoroughly examine the causative components of these associations.

It is advised that educational establishments offer structured curricula that enable learners to develop these psychological competencies. Students' psychological wellbeing will rise with interventions that specifically target the development of stress management, emotional resilience, and self-regulation abilities. It is advised that future research use experimental and longitudinal designs to more thoroughly examine the causative components of these associations.

## 6 Limitations of the study

Several limitations of the present study should be acknowledged. First, the cross-sectional design prevents causal inferences regarding the relationships among psychological flexibility, mindfulness, self-compassion, and mental health. Second, all variables were measured through self-report instruments, which may involve social desirability and common method bias. Third, the sample was obtained using convenience sampling from two universities in Istanbul, which limits the generalizability of the findings to other regions, age groups, or clinical populations. Future research should employ longitudinal and experimental designs to better examine the causal pathways suggested by the mediation model. Intervention studies testing mindfulness- and compassion-based programs within university settings would be particularly valuable. Additionally, including objective indicators of mental health and recruiting more diverse samples would strengthen external validity.

## Data Availability

The original contributions presented in the study are included in the article/supplementary material, further inquiries can be directed to the corresponding author.
